# Monkeypox: A clinical update for paediatricians

**DOI:** 10.1111/jpc.16171

**Published:** 2022-08-18

**Authors:** Yuanfei A Huang, Annaleise R Howard‐Jones, Shireen Durrani, Zhicheng Wang, Phoebe CM Williams

**Affiliations:** ^1^ National Centre for Immunisation Research and Surveillance Sydney Children's Hospital Network Sydney New South Wales Australia; ^2^ New South Wales Health Pathology Institute of Clinical Pathology and Medical Research (ICPMR) Sydney New South Wales Australia; ^3^ Faculty of Medicine and Health The University of Sydney Sydney New South Wales Australia; ^4^ School of Pharmacy, Faculty of Medicine and Health The University of Sydney Sydney New South Wales Australia; ^5^ Department of Immunology and Infectious Diseases Sydney Children's Hospital Sydney New South Wales Australia; ^6^ School of Women and Children's Health The University of NSW Sydney New South Wales Australia; ^7^ School of Public Health, Faculty of Medicine The University of Sydney Sydney New South Wales Australia

**Keywords:** ACAM2000, child health, monkeypox, MVA, stigma, vaccinia immunoglobulin

## Abstract

The global spread of human monkeypox disease, a zoonotic infection related to smallpox and endemic to West and Central Africa, presents serious challenges for health systems. As of July 2022, 14 533 cases have been reported world‐wide, leading to designation as a Public Health Emergency of International Concern. Monkeypox disease is spread from animals to humans through infected lesions or fluids; human–human transmission occurs through fomites, droplets or direct contact. Illness is usually self‐limiting, but severe disease can occur in specific groups ‐ particularly children, and people who are immunocompromised or pregnant. Clinical presentation may include fever, lymphadenopathy and skin rash, but the rash may occur without other symptoms. Complications can include secondary bacterial infection of skin lesions, vision loss from corneal involvement, pneumonia, sepsis and encephalitis. Diagnosis of monkeypox requires consideration of epidemiological, clinical and laboratory findings, with sensitive history‐taking, to elicit close contacts, critical. Supportive management is usually sufficient, but treatment options (where required) include antivirals and vaccinia immune globulin. A paucity of safety data for relevant antivirals may limit their use. There are two types of monkeypox vaccines: a replication‐competent vaccinia vaccine, the use of which is logistically and clinically complex, and a replication‐deficient modified vaccinia Ankara virus vaccine. Preparedness of health systems for addressing the current outbreak is constrained by historic underfunding for research, and compounded by stigma and discrimination against cases and affected communities. Key challenges in halting transmission include improving vaccine equity and countering discrimination against men who have sex with men to aid diagnosis and treatment.

**Key Points**

1. Monkeypox is a viral zoonotic disease that has been endemic in Central and West Africa for many decades. A global outbreak began in May 2022.
2. It is caused by monkeypox virus, a member of the Orthopoxvirus genus, which also includes the smallpox (variola) and vaccinia viruses.
3. Monkeypox is transmitted through close contact with an infected person or animal, or with material contaminated with the virus.
4. Monkeypox disease is usually self‐limiting, but severe cases can occur, especially in pregnancy, children and immunocompromised patients.
5. Smallpox vaccines can be used for pre‐exposure prophylaxis and post‐exposure prophylaxis against monkeypox disease, but are in limited supply and may be associated with serious adverse effects. An antiviral agent developed for the treatment of smallpox has also been licensed for the treatment of monkeypox.

Monkeypox is a zoonotic disease capable of human‐to‐human transmission. It is caused by the monkeypox virus, an enveloped double‐stranded DNA virus. The virus belongs to the Orthopox genus of the Poxviridae family, shared by the smallpox (variola) virus and the vaccinia virus, which is used in smallpox vaccines.^1^ Monkeypox is endemic to a number of countries in sub‐Saharan Africa, where it has caused significant morbidity and mortality for over five decades.^2^ A global outbreak beginning in May 2022 involving 72 non‐endemic countries has resulted in 14 533 probable and laboratory‐confirmed cases, alongside three deaths as of 20 July 2022.^3^

Whilst most monkeypox cases recover without severe consequences, the disease can cause significant morbidity and mortality in pregnancy, infancy and immunocompromised patients. Furthermore, monkeypox is considered a high‐consequence infectious disease, as it may be difficult to diagnose, is capable of person‐to‐person transmission and has limited effective prophylaxis and treatment options.^4^

## Historical Perspectives

The name ‘monkeypox’ stems from the virus's isolation from imported macaque monkeys in a Danish laboratory in 1958.^5^ The clinical disease was first recognised in 1970 in a 9‐month‐old male in the Democratic Republic of the Congo, who survived monkeypox but died from a subsequent measles infection.^6^ Over the following decades, sporadic cases of monkeypox were reported across Central and West Africa, mostly affecting children and resulting in a mortality rate of up to 17%.^7^, ^8^ However, the true burden and mortality rate are difficult to ascertain, given the likely under‐diagnosis and under‐reporting over prior decades when the disease primarily affected resource‐constrained regions.

Two distinct clades of the monkeypox virus have evolved, which vary in their clinical features and severity.^9^ The Central African clade is associated with more significant morbidity, mortality, transmissibility and viraemia^9^; the 2022 global outbreak is predominantly due to the West African clade, previously mostly reported in Nigeria. Since the first‐ever case of monkeypox was recognised, there have been sporadic cases and outbreaks in non‐endemic regions initiated by international travel.

**Fig. 1:**
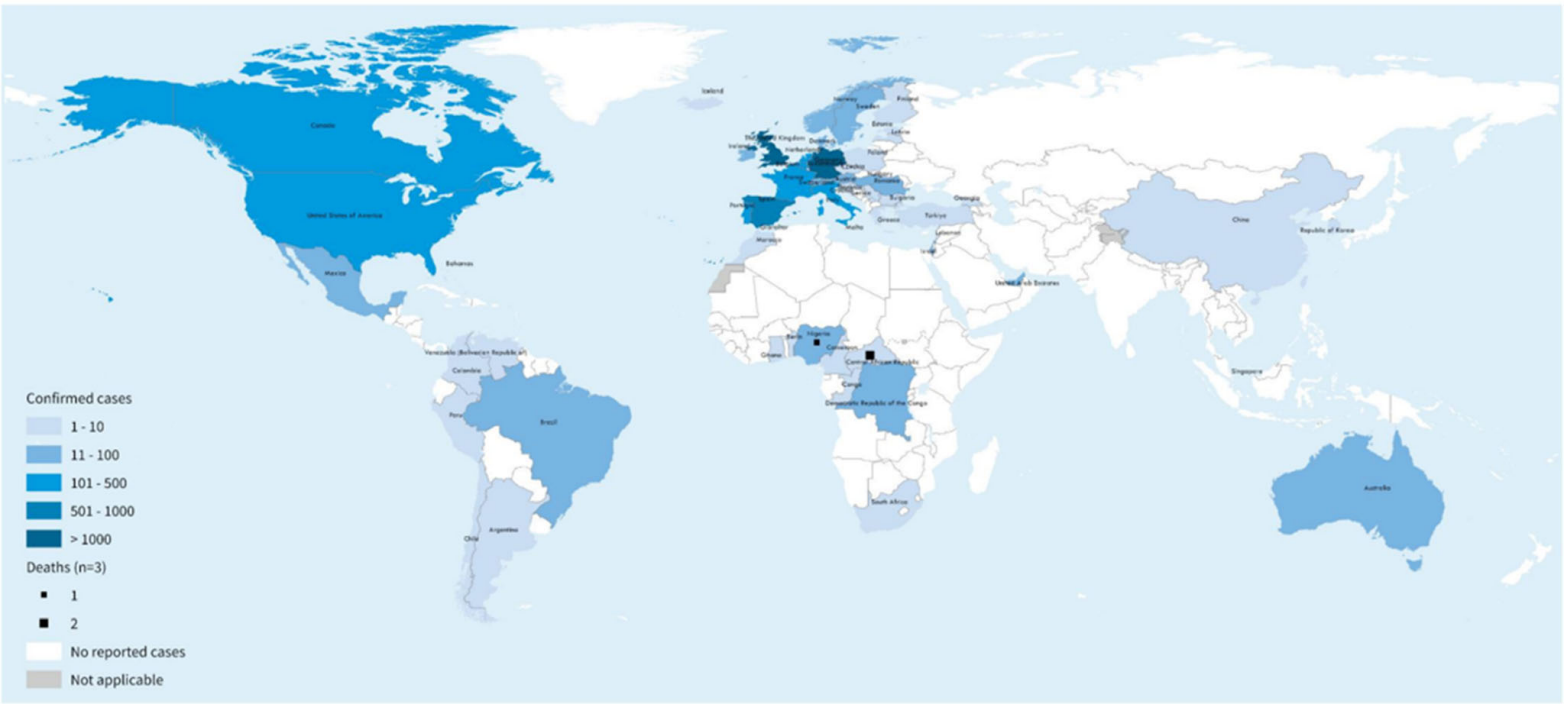
Geographic distribution of 6027 confirmed monkeypox cases reported to the World Health Organization (WHO) from 1 January to 4 July 2022. (Reproduced from World Health Organization^13^ with permission.)

When the smallpox eradication programme – which used first‐generation smallpox vaccines offering cross‐protection against monkeypox – ended successfully in 1980, the World Health Organization (WHO) established an active surveillance programme for monkeypox. This was discontinued in 1986, after predictions that future outbreaks would be self‐limiting.^10^, ^11^ However, outbreaks have occurred more frequently since 2016, attributed to reduced population immunity against smallpox, increased contact between humans and wildlife driven by urbanisation and biodiversity collapse, and improvements in surveillance systems in the wake of the Ebola epidemic.^2^, ^12^ These outbreaks received little global attention until the current outbreak that primarily affects Europe and North America, with cases detected in Australia (Fig. 1). To date, this outbreak has primarily affected men who have sex with men, although transmission into other population groups is increasing.^13^

## Transmission and Clinical Symptoms

In enzootic countries, the exact reservoir species of monkeypox are unknown, but rodents are most likely.^14^ Animal‐to‐human transmission can occur from direct contact with bodily fluids or cutaneous lesions of infected animals. This drove an outbreak of 71 cases in the United States in 2003, after pet prairie dogs were infected by African rodents whilst housed together in a distribution centre.^15^, ^16^ Human‐to‐human transmission can occur through direct contact with skin lesions or infected skin, respiratory droplets and fomites, or through the transplacental route.^17^, ^18^

**Fig. 2:**
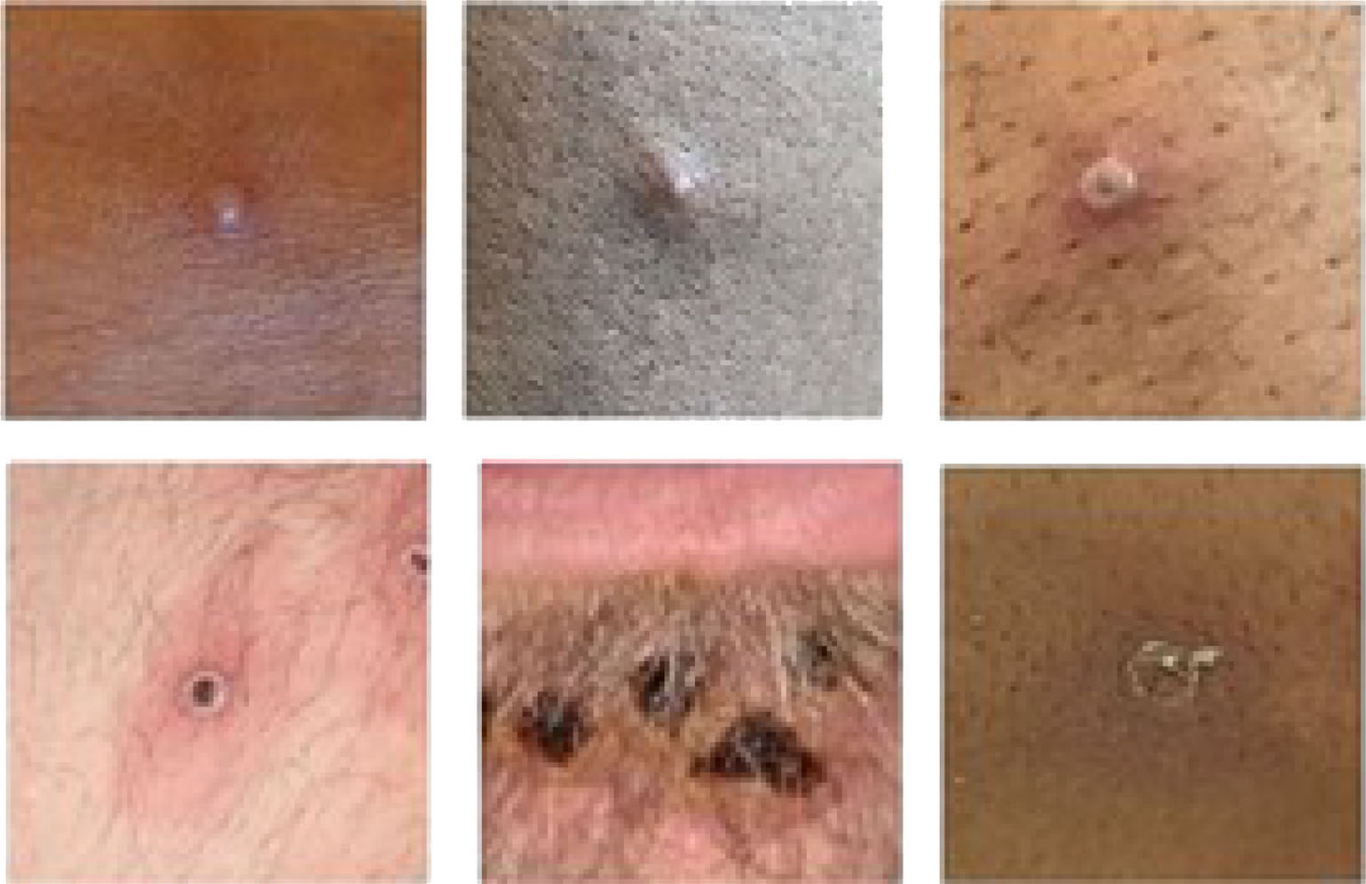
The monkeypox rash – phases of evolution through papular, vesicular and umbilicated stages before crust formation and desquamation. Photo credit: UK Health Security Agency (2022).

The clinical syndrome of monkeypox disease can be divided into two phases (Table 1).^17^ After an incubation period of 7–13 days (range 5–21 days), there is typically a 1–5 day prodromal period with fever, lymphadenopathy and influenza‐like illness preceding the onset of a rash. A skin eruption subsequently occurs, although the rash may occur without prodrome.^16^ Both the morphology and the distribution of the rash evolve over the course of illness, starting as macules and progressing over 2–3 weeks through papular, vesicular, pustular and umbilicated stages before crust formation and desquamation (Fig. 2).^17^ During this time, the rash typically moves from the facial area distally to the extremities, including the palms and soles (centrifugal spread). Involvement of oropharyngeal, anogenital or ocular mucosa can result in ulceration, which can be painful and result in dehydration and malnutrition.^17^

**Table 1:** Signs and symptoms of classical monkeypox disease^17^, ^19^

	Initial (prodromal) phase	Second phase
Common manifestations	Fever Headache Back pain Myalgia Malaise Lymphadenopathy	Evolving rash over sequential stages – macules, papules, vesicles, pustules and umbilication, prior to crusting over and desquamating over a period of 2–3 weeks (Fig. 2) Eruption tends to be centrifugal: starting on the face and progressing towards the hands and feet, and can involve the oral mucous membranes, conjunctiva, cornea and/or genitalia Observations from the 2022 outbreak describe lesion(s) commencing in the genital area (often with only a single lesion observed), and a predisposition for rash to occur without a prodromal phase
Severe manifestations		Bacterial skin and soft tissue infections (cellulitis, abscesses, necrotising soft tissue) Severe pneumonia Corneal infection which may lead to vision loss Vomiting and diarrhoea, which may result in severe dehydration, electrolyte abnormalities and shock Sepsis Encephalitis

Monkeypox is usually a self‐limiting disease with symptoms lasting from 2–4 weeks, but complications can occur in children, pregnant women and immunocompromised people.^17^ These include secondary bacterial infection of skin lesions, exfoliation of large areas of skin requiring surgical grafting, vision loss due to corneal lesions or secondary ocular infection, pneumonia, retropharyngeal abscess from cervical lymphadenopathy, sepsis and encephalitis.^16^ Patients may also suffer significant psychological distress as a result of fear, stigma and the current requirement in many jurisdictions to undergo a prolonged 21‐day period of isolation.^17^

## Disease Spectrum in Children and the Peripartum Period

Although children are often significantly affected by monkeypox,^20^ literature on the paediatric population is confined to small case series, due to limited epidemiological surveillance and poor access to diagnostic tools in resource‐limited settings.^12^ Monkeypox in children is usually acquired in the household or following close contact with infected animals.^21^ Particular risk factors include cohabiting the same bed or room, or sharing utensils with an infected person.^21^ As the disease has spread throughout the UK in 2022, increasing numbers of children have been exposed within households.^22^

To date, children aged under 10 years represent a large proportion of monkeypox‐related deaths, ranging from 100% of documented fatalities in the period 1970–1999 to 37.5% over 2000–2019.^1^ In children, presentation with oropharyngeal monkeypox lesions and nausea or vomiting has been associated with extended hospitalisation.^20^ Severe manifestations – including pneumonitis, corneal ulceration, encephalitis, multi‐organ failure and (rarely) fulminant hepatosplenic infiltration – have been reported in infants and young children and have the potential to cause death or long‐term disability.^23^, ^24^, ^25^, ^26^ Clinical manifestations may be indistinguishable from varicella zoster virus (VZV) infection^27^ and coinfection with VZV is well recognised.^28^, ^29^

Congenital monkeypox infection has been described in one case series from the Democratic Republic of the Congo, with stillbirth a tragically common outcome.^18^, ^30^ Three of the four infections described in this case series occurred after maternal infection in the first and second trimester, and resulted in fetal demise.^18^ Vertical transmission was confirmed in one fetus, presenting with elevated viral loads in fetal tissues, disseminated cutaneous lesions, hepatomegaly and hydrops fetalis.^18^

Recent European and UK guidance issued for the diagnosis and management of suspected monkeypox in pregnant women include the potential utility of available antivirals, vaccinia immune globulin (VIG) and safety considerations of post‐exposure vaccination.^31^, ^32^ Although the optimal mode of delivery to minimise overall transmission is unclear, caesarean section is recommended if genital lesions are present.^32^ Infants born to mothers with monkeypox infection during pregnancy should be monitored closely in a special care unit.^31^ Sampling of eye, skin and mouth (alongside swabs from skin lesions) and sampling of infant or umbilical cord blood for monkeypox‐specific polymerase chain reaction (PCR) testing is recommended.^31^

## Diagnosis

**Fig. 3:**
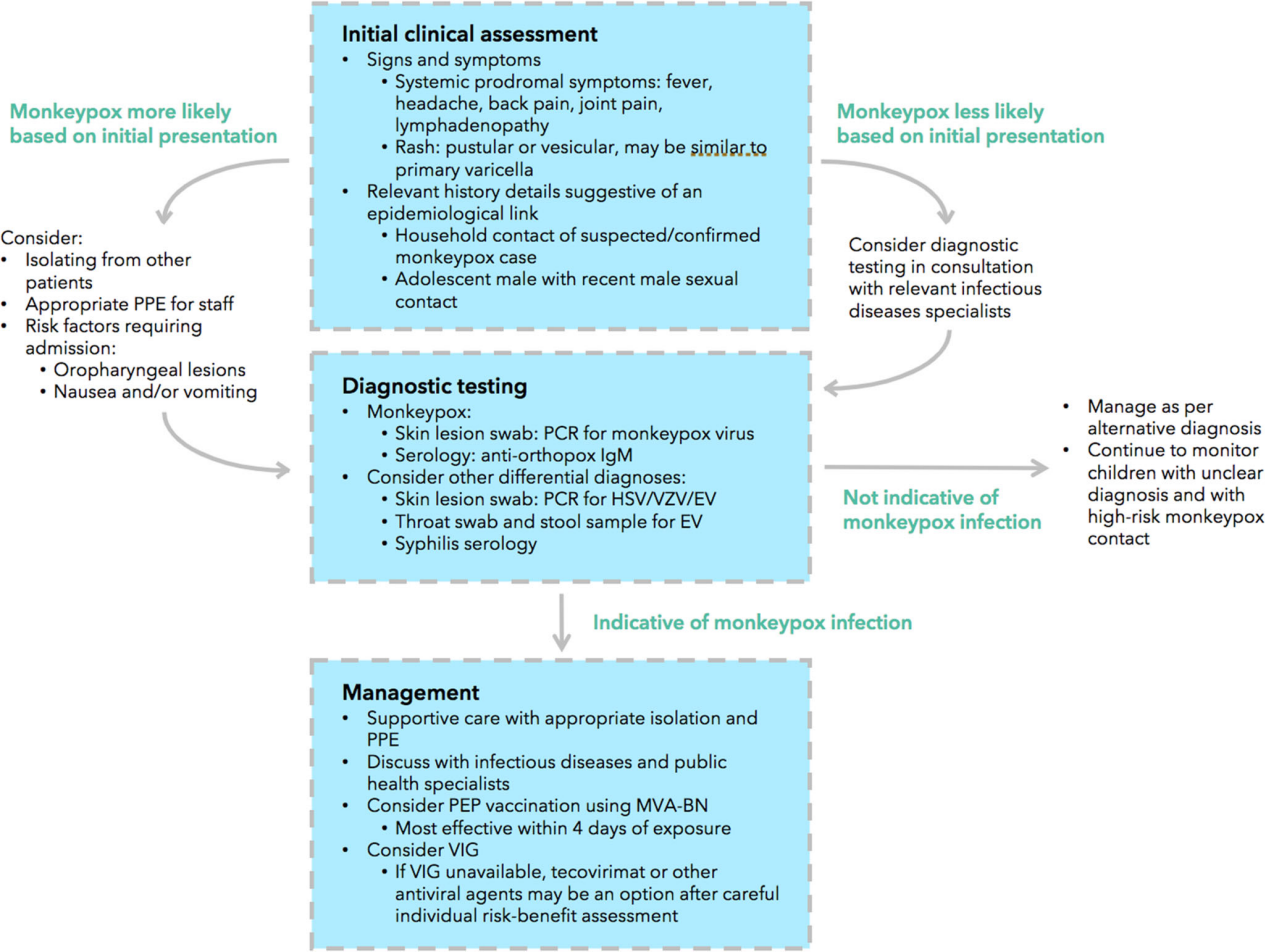
Key features pertaining to the diagnosis and management of monkeypox disease. EV, eczema vaccinatum; HSV, Herpex simplex virus; MVA‐BN, modified vaccinia Ankara virus vaccine; PCR, polymerase chain reaction; PEP, post‐exposure prophylaxis; PPE, Personal protective equipment; VZV, varicella zoster virus. Source: Original figure by authors (2022).

The diagnosis of monkeypox should consider the epidemiological, clinical and (if available) laboratory findings.^33^ Clinical cases may be classified as suspected, probable or confirmed, guided by any history of exposure to monkeypox within the last 21 days (Fig. 3).^34^ Ascertaining a history of close contact with suspected monkeypox cases requires a sensitive approach given the significant stigma surrounding the condition.^35^ Children who have had household‐like contact with a case warrant careful monitoring. If a compatible rash evolves, children should undergo prompt PCR testing from samples of the lesion (at a laboratory designated for orthopox monitoring) alongside testing for other causes of vesicular rash (including herpes simplex virus, VZV, enterovirus and syphilis). Serological testing can also be used, with monkeypox patients typically having detectable levels of anti‐orthopox IgM antibody 4–56 days after rash onset; or a four‐fold rise in IgG antibody titre between acute and convalescent (day 21 onwards) samples. Histopathological analysis of skin lesions is not generally helpful, as findings are non‐specific.^36^

The WHO recommends that health‐care workers looking after patients with suspected or confirmed monkeypox should observe contact and droplet precautions and wear appropriate personal protective equipment. If aerosol‐generating procedures are performed or varicella infection is suspected, the patient should also be cared for with airborne precautions.^17^ There is ongoing research to clarify the potential for short‐range aerosol transmission.^17^ For probable or confirmed monkeypox cases, timely referral to local public health authorities is critical for effective contact tracing, isolation and preserving the potential to prevent endemicity.

## Limitations of Available Vaccines

Smallpox vaccines using vaccinia virus are stockpiled due to the potential threat of smallpox bioterrorism, and these can also be used against monkeypox, for either pre‐exposure prophylaxis (PrEP) or post‐exposure prophylaxis (PEP). Evidence for the effectiveness of vaccinia vaccines specifically against monkeypox has been largely indirect, drawing on *in vitro* and challenge study data in animals.^11^

There are two types of smallpox vaccines:

1. A live attenuated replication‐competent vaccinia vaccine (proprietary name ACAM2000), which is logistically challenging to administer, requiring percutaneous scarification using a bifurcated needle by trained vaccinators (for whom pre‐exposure vaccination to recommended to protect against inadvertent exposure to live vaccine virus);^37^
2. A replication‐deficient modified vaccinia Ankara virus vaccine (MVA‐BN; trade names Jynneos/Imvanex/Imvamune) which is currently registered in North American and European countries for the prevention of smallpox and monkeypox.

ACAM2000 is currently available in Australia for PrEP and PEP against monkeypox.^37^ However, as a live, replication‐competent vaccine, it is contraindicated for immunocompromised people, pregnant and breastfeeding women and children under 12 months of age. ACAM2000 has the potential to cause a range of other serious adverse events associated with first‐generation smallpox vaccines. These include autoinoculation: the transfer of vaccine virus, usually through scratching (particularly in children) from the vaccination site to other areas, where a rash may develop.^38^ Autoinoculation of the eye can cause vaccinial keratitis, which may lead to permanent vision loss,^39^ and autoinoculation of areas with active atopic dermatitis can result in the potentially fatal syndrome eczema vaccinatum.^38^ Other manifestations of vaccine‐derived vaccinia dissemination include generalised vaccinia (a widespread exanthem resulting from viraemia that is usually transient in immunocompetent vaccines) and progressive vaccinia (more frequently seen in immunocompromised individuals, where skin lesions radiate from the inoculation site, leading eventually to widespread cutaneous and systematic disease that may be fatal). Dissemination of the vaccinia virus to the central nervous system can result in post‐vaccinial encephalopathy or post‐vaccinial encephalomyelitis. Finally, transplacental transmission of vaccinia virus can occur in women vaccinated during pregnancy, leading to fetal vaccinia and potentially stillbirth or perinatal death.^18^

By contrast, as the MVA‐BN vaccine is replication‐deficient (the live virus has been attenuated such that it is unable to replicate within cells), it is theoretically considered to be a safe vaccine for children, pregnant women and immunocompromised patients. Administration is by subcutaneous injection, which precludes autoinoculation with vaccine virus at other body sites. No serious safety concerns were identified during clinical trials in people receiving at least one dose of MVA‐BN, except one case of possible vaccine‐related pericarditis.^40^, ^41^ In the UK, the MVA‐BN vaccine is the mainstay for both PrEP and PEP vaccination in the 2022 monkeypox outbreak; in light of limited supply, the PrEP vaccination strategy targets those at the highest risk of monkeypox infection.^42^ In countries where it is available, PEP vaccination with MVA‐BN is prioritised for those at the highest risk of complications, such as children, pregnant women and people with immunosuppression.

## Limitations of Current Treatment Options

Most cases of monkeypox are self‐limiting, with only supportive management needed. Hospital admissions in the current outbreak have occurred primarily to facilitate required isolation periods, manage severe anorectal or oropharyngeal pain, treat soft tissue superinfection, or administer therapies for severe disease.^19^

Treatments for monkeypox infections include antiviral agents and VIG, all of which are considered investigational for this indication.^40^ The only orthopoxvirus‐specific antiviral available is tecovirimat, an oral medication for smallpox.^43^ The safety of tecovirimat has been assessed in only one human phase I trial, meaning that its adverse effect profile remains unknown, including its safety in children and pregnancy.^43^

Cidofovir is an antiviral agent registered for the treatment of cytomegalovirus in immunocompromised patients that has also been used against adenovirus and human herpesvirus.^40^ Cidofovir has *in vitro* activity against monkeypox,^44^ but its side effect profile (including nephrotoxicity) limits its use. Brincidofovir, an oral analogue of cidofovir, has limited published data on use in monkeypox but also has a significant side effect profile: when used in the treatment of three adult monkeypox cases in the UK in 2018, all patients developed an elevation in alanine aminotransferase levels that led to its discontinuation.^4^

Finally, VIG – an intramuscular preparation of hyperimmune globulin prepared from the pooled blood of individuals vaccinated with smallpox vaccine – may be considered in immunosuppressed patients exposed to monkeypox, in whom ACAM2000 vaccine is contraindicated. VIG may also be used for aberrant infections induced by vaccinia virus due to autoinoculation, eczema vaccinatum, progressive vaccinia, or severe generalised vaccinia.^45^ The current Australian guidelines reserve VIG as a second‐line treatment for monkeypox infection, if tecovirimat is unavailable.^40^ VIG may also be considered for pregnant women, children and immunocompromised people, given the lack of safety data for tecovirimat.

## Stigma, Discrimination and Inequity

Although monkeypox can infect all humans, concerns about stigma and discrimination associated with the current transmission of monkeypox in men who have sex with men are at the forefront of control measures and public communication. In many countries, under‐diagnosis of monkeypox is likely in the context of non‐heterosexual orientations being criminalised.^46^ This should be a priority consideration after the designation of monkeypox as a Public Health Emergency of International Concern, as it may legitimise government persecution for sexual orientation.^3^

Other forms of discrimination have included global inequities in access to vaccines, antiviral agents and laboratory testing capacity.^47^ The MVA‐BN vaccine has been made available to very limited countries outside Europe and North America, and many potential cases of monkeypox in less‐resourced countries remain a ‘suspected’ classification due to a lack of PCR testing capacity. Whilst global efforts to share information through open‐source channels have been laudable, most viral sequences on genomic sharing websites have originated from laboratories in Europe and North America. Data on confirmed cases have been compiled on the GitHub website using public domain sources, but clinical details have only been published in‐depth for cases in high‐income countries.^48^ A colonial undertone remains in much of the global discourse on monkeypox,^49^ and the push by the WHO to rename the virus and its clades are necessary steps in reducing the stigma and discrimination associated with the disease.^50^

## Conclusion

Monkeypox is a high‐consequence infectious disease that, despite decades of endemicity in sub‐Saharan Africa, has only received widespread attention in 2022 with the emergence of a global outbreak affecting over 70 countries. Due to limited research into transmission, clinical severity and treatment options, the international community is poorly placed to prevent and treat this zoonotic disease despite ongoing human‐to‐human transmission and likely increased animal‐to‐human transmission, in the face of a changing global epidemiological climate. Alongside these challenges, the stigma and discrimination currently borne by patients infected with monkeypox impacts case ascertainment, and places vulnerable communities at increased risk of poor disease outcomes and continued transmission.
